# *Megasphaera elsdenii*: from gastrointestinal functional symbiont to industrial biomanufacturing chassis

**DOI:** 10.3389/fmicb.2026.1860116

**Published:** 2026-06-17

**Authors:** Ziyun Li, Li Zhang, Yueke Lin, Huijuan Liu, Ting Zhang, Yan Li

**Affiliations:** 1 Shandong Provincial Maternal and Child Health Care Hospital Affiliated to Qingdao University, Qingdao University, Jinan, China; 2 Department of Clinical Laboratory, The Second Qilu Hospital of Shandong University, Shandong University, Jinan, China

**Keywords:** biosafety risk, industrial biomanufacturing chassis, lactate metabolism, *Megasphaera elsdenii*, probiotic potential

## Abstract

Anaerobic gastrointestinal commensals are critical regulators of host health and key chassis resources for sustainable green biomanufacturing. *Megasphaera elsdenii* is a Gram-negative, strictly anaerobic coccus that widely colonizes the gastrointestinal tract of mammals, especially the rumen of ruminants. Its defining metabolic feature is the highly efficient catabolism of lactate via the acrylate pathway, coupled with the synthesis of short- and medium-chain fatty acids (SCFAs/MCFAs), biohydrogen, and high-value metabolic intermediates. This review systematically compiles the fundamental biological characteristics, core metabolic networks, and host-specific physiological functions of *M. elsdenii* across ruminants, humans, and non-ruminant mammals. We highlight its dual nature: as a promising probiotic candidate for preventing ruminal acidosis and maintaining intestinal homeostasis, and as a high-potential anaerobic chassis strain for biomanufacturing organic acids, bioenergy, and bioplastic precursors. Meanwhile, we comprehensively discuss its biosafety risks, including conditional pathogenicity, antibiotic resistance gene dissemination, and gut microecological disturbance, as well as unresolved scientific controversies. Finally, we identify critical research gaps and propose future research priorities, providing a systematic framework for the rational development and safe application of this microbe.

## 1 Introduction

*Megasphaera elsdenii* is a Gram-negative, strictly anaerobic coccus belonging to the genus *Megasphaera* of the family Veillonellaceae, phylum Firmicutes, whose taxonomic status has been firmly validated by 16S rRNA gene sequencing analysis and genomic characterization (Sugihara et al., 1974; Mamphogoro et al., 2025). This strain was first isolated and identified from the rumen contents of ruminants in 1953, initially classified into the genus *Peptostreptococcus*, and later reassigned to the genus *Megasphaera* following taxonomic revisions (Cabral and Weimer, 2024). Morphologically, it predominantly occurs in pairs with a typical diameter of approximately 1.5 μm, encased in a thick fibrous carbohydrate capsule extracellularly, and its cell wall contains characteristic plasmalogen components (Costerton et al., 1974; Kaufman et al., 1990; Cabral and Weimer, 2024). Physiologically, it has an absolute requirement for anaerobic conditions for growth, with core metabolic traits of high-efficiency lactate utilization capacity and medium-chain fatty acid (MCFA) synthetic potential, which serve as the key discriminatory features distinguishing it from other species within the same genus (Shetty et al., 2013; Strachan et al., 2025).

As a key functional bacterium in the mammalian gastrointestinal ecosystem, the research value of *M. elsdenii* spans multiple fields including microbial ecology, animal nutrition, human health, and industrial biotechnology. In the field of host health regulation, this bacterium is the dominant microbial group responsible for ruminal lactate metabolism in ruminants, contributing to 60–80% of total lactate degradation in the rumen. It converts lactate into short-chain fatty acids (SCFAs) such as propionate via the acrylate pathway, thereby effectively maintaining ruminal pH homeostasis and acting as a core functional bacterium for the prevention and control of ruminal acidosis (Counotte and Prins, 1981; Susanto et al., 2023). In monogastric animals, it modulates intestinal lactate metabolic balance, promotes SCFAs production, improves intestinal mucosal development, and reduces the incidence of diarrhea (Muya et al., 2015; Yoshikawa et al., 2018). As a resident member of the human intestinal microbiota, *M. elsdenii* participates in SCFAs synthesis and intestinal homeostasis regulation. Alterations in its abundance are closely associated with multiple diseases including inflammatory bowel disease (IBD), colorectal cancer (CRC), and diabetes mellitus, yet significant controversies remain regarding its probiotic versus pathogenic roles in different intestinal disorders (Sugihara et al., 1974; Hou et al., 2025; Wang et al., 2025).

In the field of industrial applications, the metabolic diversity of *M. elsdenii* endows it with promising developmental potential as a chassis strain for synthetic biology. It can synthesize MCFAs such as caproic acid and valeric acid from a wide range of substrates via the reverse β-oxidation pathway, providing core precursors for the production of biofuels and industrial chemical feedstocks (Choi et al., 2013, p. 702410; Lee et al., 2020). Its lactate-driven dark fermentative hydrogen production trait offers a novel solution to the intractable problem of lactate accumulation-mediated inhibition in biological hydrogen production, and relevant functional enzymes derived from this bacterium have been applied to the metabolic engineering construction of biopolymers (Zhang et al., 2019; Ohnishi et al., 2022). In addition, the transmission risk of tetracycline-class chimeric resistance genes carried by *M. elsdenii*, as well as its opportunistic pathogenicity under rare circumstances, also provide important research targets for antimicrobial resistance (AMR) control and pathogenic mechanism studies (Brancaccio and Legendre, 1979; Stanton and Humphrey, 2003; Stanton et al., 2004).

With the advancement of high-throughput sequencing, multi-omics, and synthetic biology technologies, research on *M. elsdenii* has evolved from basic physiological and biochemical characterization to in-depth dissection of genomic function, host–microbe interaction mechanisms, and applied technology development. However, critical research gaps remain: the causal mechanisms underlying its association with human diseases have not been validated by clinical intervention studies; low product tolerance and high process costs in industrial fermentation restrict its industrial translation; the transmission mechanisms of antibiotic resistance genes (ARGs) and regulatory factors of pathogenicity are still poorly understood. This review systematically summarizes the biological characteristics, metabolic mechanisms, physiological functions, and application potential of *M. elsdenii*, and dissects its potential risks and unresolved research controversies. It aims to integrate multidisciplinary research findings, define the current boundaries of research in this field, and provide a comprehensive academic reference for the in-depth exploration and rational utilization of this bacterium.

## 2 Basic biological characteristics

### 2.1 Morphological and cultural characteristics

*M. elsdenii* is a Gram-negative, strictly anaerobic coccus with a typical cell diameter of approximately 1.5 μm, predominantly occurring in pairs and occasionally in short chains (Costerton et al., 1974; Kajihara et al., 2017). Its thick extracellular fibrous carbohydrate capsule is closely associated with gastrointestinal colonization ability (Costerton et al., 1974). The cell wall is rich in plasmalogen components, with a core skeleton consisting of phosphatidylethanolamine and phosphatidylserine, which serves as a key taxonomic marker to distinguish it from other genera in the family Veillonellaceae (van Golde et al., 1973; Kaufman et al., 1990). Notably, the plasmalogen content of some strains decreases significantly after serial subculture, with no observable changes in cell morphology or growth rate (Kaufman et al., 1988).

This bacterium is extremely sensitive to oxygen and requires a strictly anaerobic culture system, with optimal growth conditions at pH 5.5–7.5 and 37 °C. Growth and metabolic activity are significantly inhibited under extreme pH conditions or when the temperature is outside the range of 25–45 °C (Vervaeke and Van Assche, 1975; Jeon et al., 2017; Kajihara et al., 2017; Fan et al., 2022; Mamphogoro et al., 2025). For nutritional requirements, lactate or glucose serves as its core carbon source, and it can utilize ammonium salts and complex protein hydrolysates as nitrogen sources. Its growth is dependent on B vitamins including biotin, and cysteine is the optimal sulfur source as well as a reducing agent (Forsberg, 1978; Kajihara et al., 2017). It is commonly isolated using Kajihara-Megasphaera-Isolation (KMI) selective medium, forming typical large, yellowish-white, smooth-surfaced colonies with a diameter of 2–3 mm (Kajihara et al., 2017).

### 2.2 Genomic and genetic characteristics

To date, whole-genome sequencing (WGS) has been completed for multiple *M. elsdenii* strains. Its genome size ranges from 2.5 to 2.8 Mb, with a G + C content of 51.8–52.1%, a core gene region accounting for approximately 85% of the genome, and mobile genetic elements (MGEs) accounting for 3–5% (Shetty et al., 2013; Hatmaker et al., 2019, p. 25940; Lee et al., 2020; Mamphogoro et al., 2025). The 16S rRNA gene sequence similarity among strains from different host sources reaches 98.9–99.2%, and phenotypic differences are mainly derived from substrate utilization preferences and metabolite production ratios (Shetty et al., 2013; Kajihara et al., 2017; Yoshikawa et al., 2018). The differentiation of core carbohydrate-active enzyme (CAZyme) families between human-derived and rumen-derived strains reflects adaptive evolution to the host’s dietary environment.

Functional genomic studies of this bacterium have mainly focused on lactate metabolism and fatty acid synthesis pathways. Its lactate metabolism-related genes mainly encode D-lactate dehydrogenase (D-LDH), lactate racemase (LR), and key enzymes of the acrylate pathway. Among them, D-LDH is constitutively expressed, while LR is only expressed under lactate induction (Hino and Kuroda, 1993; Prabhu et al., 2012). The propionyl-CoA transferase gene (*pct*) is involved in the conversion of lactate to propionate, and its heterologous expression can significantly enhance the propionate synthesis capacity of the host strain (Zhang et al., 2019). This bacterium universally harbors fatty acid synthesis-related genes, including those encoding enzymes for SCFA synthesis (acetyl-CoA acetyltransferase, 3-hydroxyacyl-CoA dehydrogenase) and key genes for MCFA synthesis (genes encoding enzymes involved in the reverse β-oxidation pathway) (Choi et al., 2013, p. 702410; Lee et al., 2020). In addition, plasmalogens are a class of glycerophospholipids with a unique vinyl ether bond structure. The plasmalogen synthase gene (*MeHAD*, MELS_0169) in *M. elsdenii* is encoded by a single gene, and heterologous expression of this gene in *Escherichia coli* can significantly improve the oxidative stress resistance and osmotic tolerance of the host strain (Zhang et al., 2023).

Additional functional genes have also been identified in *M. elsdenii*, including those encoding amino acid deaminase (involved in branched-chain amino acid degradation) and components of the phosphoenolpyruvate-dependent phosphotransferase system (PTS), which is responsible for the transport and phosphorylation of glucose and fructose (Dills et al., 1981; Rychlik et al., 2002). Some strains carry the phytase gene (*phyAme*), which can degrade phytic acid to release inorganic phosphorus (Yanke et al., 1998). Notably, studies led by Stanton *et al.* have demonstrated that tetracycline resistance genes are universally detected in *M. elsdenii* strains, with associated horizontal transmission risks. Strains isolated from swine feces carry chimeric tetracycline resistance genes *tet(O)* and *tet(W)*, forming at least 7 genotypes, with minimum inhibitory concentrations (MICs) of these strains against tetracycline antibiotics ranging from 128 to >256 μg/mL (Stanton and Humphrey, 2003; Stanton et al., 2004). The abundance of tetracycline-resistant *M. elsdenii* strains in the intestinal tract of organically raised pigs is significantly higher than that in wild pigs, suggesting that antibiotic selective pressure in the farming environment may drive the spread of ARGs (Stanton et al., 2011).

### 2.3 Genetic manipulation toolkit for *Megasphaera elsdenii*

As an emerging industrial biomanufacturing chassis strain, *M. elsdenii* currently lacks a mature native genetic manipulation system. To date, no validated protocols for natural transformation, conjugation, or electroporation have been established for wild-type *M. elsdenii* strains, and the low transformation efficiency of existing tentative methods fails to meet the requirements of synthetic biology. Notably, all reported genetic modifications of *M. elsdenii* functional proteins rely on site-directed mutagenesis following heterologous expression in model strains (Nguyen et al., 2026). Current research on the functional genes of this bacterium predominantly depends on heterologous expression in model strains (e.g., *E. coli*) instead of native genetic modification of *M. elsdenii* (Li et al., 2016; Zhang et al., 2019, 2023). The absence of native genetic tools severely restricts the targeted engineering of its metabolic pathways and the optimization of its industrial performance.

## 3 Core metabolic mechanisms

The metabolic network of *M. elsdenii* is characterized by the efficient conversion of carbon sources, with core pathways constructed around the catabolism of substrates such as lactate and glucose and fatty acid anabolism, supplemented by auxiliary metabolic processes including amino acid conversion and specialized lipid biosynthesis. Its metabolic flexibility is the fundamental basis for the bacterium’s adaptation to the gastrointestinal environment of diverse hosts and its promising industrial development potential.

### 3.1 Carbon source metabolism and energy generation

**Figure 1:**
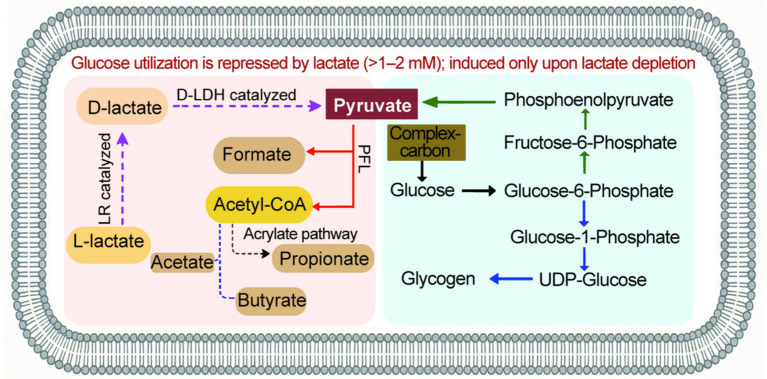
Carbon source metabolism schematic.

*M. elsdenii* exhibits significant substrate utilization preference and environmental adaptability for carbon sources, and core carbon source metabolism is directly coupled to energy production and the regulation of host physiological functions. Lactate is the preferred carbon source for this bacterium, and its efficient conversion via the acrylate pathway is the core mechanism by which it regulates pH homeostasis in anaerobic environments such as the rumen (Counotte and Prins, 1981; Prabhu et al., 2012). During metabolism, D-lactate is directly converted to pyruvate by constitutively expressed D-LDH (Figure 1), while L-lactate must be converted to D-lactate by inducible LR before entering the downstream pathway (Hino and Kuroda, 1993). Pyruvate is further converted to acetyl-CoA and formate via pyruvate-formate lyase, and acetyl-CoA is subsequently used to generate propionate through the acrylate pathway, coupled with simultaneous adenosine triphosphate (ATP) synthesis. This pathway is highly sensitive to environmental pH: the lactate degradation rate decreases significantly at pH below 5.0, while lactate concentrations in the range of 10–100 mM have a negligible effect on the metabolic rate (Chen et al., 2019; Fan et al., 2022). The end products of lactate fermentation are dominated by propionate and butyrate, with propionate accounting for 30–40% of the total products. This enables the bacterium to rapidly degrade lactate produced by acidogenic bacteria and alleviate the progression of ruminal acidosis (Arce-Cordero et al., 2023; Susanto et al., 2023; Strachan et al., 2025).

Glucose is a secondary carbon source for the bacterium. After being transported and phosphorylated via the phosphoenolpyruvate-dependent PTS, glucose is converted to pyruvate through the glycolytic pathway, which is further fermented to produce SCFAs such as acetate and butyrate. Some strains can convert more than 60% of uptaken glucose into intracellular glycogen for storage (Dills et al., 1981; Hino and Kuroda, 1993; Martin and Wani, 2000; Weimer and Digman, 2013). Certain strains can utilize complex carbon sources such as xylose and cellobiose, and follow a strict hierarchical order of substrate utilization in a mixed carbon source system: lactate is preferentially utilized, and glucose metabolism is initiated only when the lactate concentration drops below 1–2 mM (Hino et al., 1994; Shetty et al., 2013; Ohnishi et al., 2022) (Figure 1). This trait is determined by the specific regulation of the carbon source transport system.

### 3.2 Fatty acid synthesis and metabolism

*M. elsdenii* is one of the few intestinal anaerobic bacteria capable of simultaneous synthesis of SCFAs and MCFAs, and its fatty acid synthesis pathway has significant industrial application value (Choi et al., 2013; Lee et al., 2020). The synthesis of SCFAs (acetate, propionate, butyrate, valerate) is directly coupled to carbon source metabolism and is the core accompanying process of energy production. Acetate is catalyzed by acetyl-CoA synthetase, accounting for 20–30% of the total fatty acid production (van Golde et al., 1973; Susanto et al., 2023). Propionate synthesis is dominated by the acrylate pathway, with the succinate pathway as a secondary route; the switch between the two pathways is regulated by environmental pH, with the acrylate pathway being absolutely dominant at pH > 6.0 (Fan et al., 2022; Strachan et al., 2025). Butyrate and valerate are generated via the condensation of acetyl-CoA and the condensation of branched-chain amino acid metabolites with acetyl-CoA, respectively, and their yields increase significantly when lactate is used as the sole carbon source (Rychlik et al., 2002; Yoshikawa et al., 2018).

The synthesis of MCFAs such as caproic acid and heptanoic acid via the reverse β-oxidation pathway is a core metabolic feature that distinguishes *M. elsdenii* from most other intestinal anaerobes (Choi et al., 2013; Lee et al., 2020). This pathway uses short-chain acyl-CoAs such as acetate and butyrate as primers, and the carbon chain is gradually extended to 6–7 carbon atoms catalyzed by key enzymes including acyl-CoA ligase and 3-hydroxyacyl-CoA dehydrogenase, with caproic acid as the main end product (Kwon et al., 2018; Wu et al., 2019). MCFAs synthesis is highly dependent on the supply of SCFA precursors: the addition of acetate can increase caproic acid production by 30–50% (Choi et al., 2013; Kim et al., 2020), and the maximum caproic acid yield can reach 28.42 g/L in an optimized two-phase extractive fermentation system (Choi et al., 2013).

### 3.3 Other metabolites and functions

In addition to core carbon metabolism and fatty acid synthesis, the bacterium can generate branched-chain fatty acids such as isobutyric acid and isovaleric acid through the deamination of branched-chain amino acids including leucine and isoleucine, with yields positively correlated with the protein content of the host diet (Wallace, 1986; Rychlik et al., 2002). Meanwhile, *M. elsdenii* is one of the few intestinal anaerobes capable of synthesizing plasmalogens, which are catalyzed by the single-gene encoded MeHAD enzyme (Kaufman et al., 1990; Zhang et al., 2023). This component accounts for more than 75% of the total phospholipids in the cell membrane, and can significantly improve the cell’s resistance to oxidative stress and osmotic tolerance (van Golde et al., 1973; Kaufman et al., 1988; Zhang et al., 2023).

In addition, the bacterium produces CO_2_ and H_2_ during fermentation, making it an important contributor to gastrointestinal gas metabolism (Rose et al., 2021; Mutuyemungu et al., 2024). The gas production rate is significantly higher during fermentation of substrates rich in indigestible carbohydrates such as sweet potato and legumes than during monosaccharide fermentation (Mutuyemungu et al., 2024). In human fecal microbiota, communities containing *M. elsdenii* have a total gas production more than 30% higher than those without the bacterium (*p* < 0.001), which is closely related to the cross-feeding of acetate (Mutuyemungu et al., 2024). The lactate-driven dark fermentative hydrogen production trait catalyzed by its [FeFe]-hydrogenase, with which co-culture of *M. elsdenii* and lactic acid bacteria achieves stable hydrogen production with a yield of 0.95–1.49 mol H_2_ per mole of glucose in a lactate-driven dark fermentation system (Ohnishi et al., 2022), provides a novel solution to the challenge of lactate accumulation-mediated inhibition in biological hydrogen production.

## 4 Physiological functions and roles in hosts

**Figure 2:**
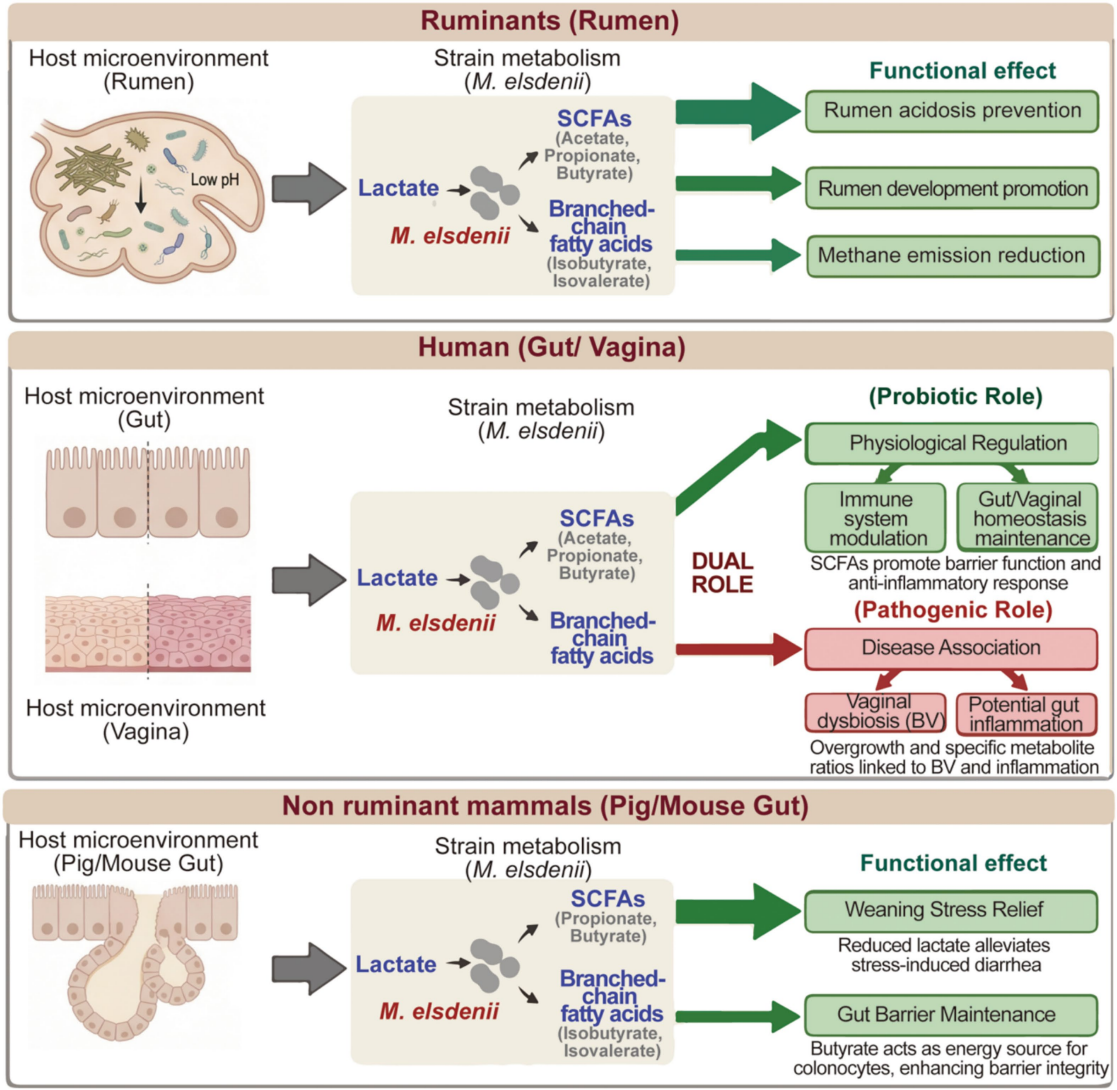
Physiological function map of *M. elsdenii* across different hosts.

As a core symbiotic bacterium in the gastrointestinal tract of mammals, *M. elsdenii* exhibits significant host specificity and environmental adaptability in its physiological functions (Figure 2). It exerts effects on host health and production performance mainly through metabolic regulation, maintenance of microecological balance, and immunomodulation, while displaying opportunistic pathogenicity under specific conditions, with its specific effects highly dependent on the host species and intestinal microenvironment.

### 4.1 Core functions in ruminants

*M. elsdenii* is a key functional bacterium in the ruminal microecology of ruminants, with its core roles focusing on the regulation of ruminal fermentation and health maintenance, which are directly linked to animal production performance. As the dominant lactate-utilizing bacterium in the rumen, it can rapidly degrade lactate produced by acidogenic bacteria such as streptococci, preventing the ruminal pH from dropping below 5.0 and significantly reducing the risk of subacute and acute ruminal acidosis (SARA and ARA) (Chen et al., 2019; Arce-Cordero et al., 2023). Under acidic conditions with pH 5.5–6.0, it maintains lactate degradation activity by upregulating the expression of key lactate metabolism genes including D-LDH and LR encoding genes, thereby stabilizing the ruminal fermentation environment (Fan et al., 2022; Susanto et al., 2023). Combined use with *Saccharomyces cerevisiae* can further increase propionate production and enhance the alleviating effect on acidosis (Monteiro et al., 2022; Khorasani et al., 2023). SCFAs such as propionate and butyrate produced by its metabolism provide the core energy source for rumen epithelial cells, promote the growth of rumen papillae and the expansion of absorptive surface area, and improve nutrient absorption efficiency (Figure 2). In neonatal calves, supplementation with this bacterium significantly increases the production of ruminal volatile fatty acids (VFAs) and accelerates the functional maturation of the rumen (Muya et al., 2015; DeClerck et al., 2020; Mazon et al., 2025). Meanwhile, branched-chain fatty acids generated by its metabolism can provide growth factors for other ruminal microorganisms and optimize the microecological structure (Wallace, 1986; Rychlik et al., 2002).

In terms of production performance, supplementation with this bacterium improves the average daily gain (ADG), hot carcass weight, and body condition score of ruminants, and reduces the incidence of diarrhea and ruminal bloat (DeClerck et al., 2020; Susanto et al., 2023). It also reduces methane emissions by 10–15% through competitive utilization of substrates for methanogens (Direkvandi et al., 2021; Li et al., 2021). However, its effect on the milk production performance of dairy cows remains controversial: some studies have indicated that increased abundance of this bacterium is associated with milk fat depression (MFD) (Weimer and Moen, 2013; Cabral and Weimer, 2024), while the specific mechanism has not yet been elucidated.

### 4.2 Physiological roles in human hosts

Although *M. elsdenii* has a low abundance in the human intestinal tract, it is extensively involved in intestinal fermentation metabolism and immunomodulation, with abnormal abundance closely linked to the occurrence and progression of multiple diseases. Physiologically, this bacterium produces SCFAs such as butyrate and valerate by fermenting intestinal lactate and indigestible carbohydrates, which supply energy to colonic epithelial cells and maintain intestinal barrier integrity (Hashizume et al., 2003; Huertas-Díaz et al., 2025). Meanwhile, as a key strain involved in acetate cross-feeding, it can utilize intestinal acetate to produce butyrate and gas, with its abundance significantly and positively correlated with the fermentation efficiency of foods such as legumes and sweet potatoes (Rose et al., 2021; Mutuyemungu et al., 2024). Recent large-scale metaproteomic studies further demonstrate that *M. elsdenii* contributes to glucose homeostasis by enhancing butyrate production, and its abundance is selectively enriched by metformin and acarbose in patients with type 2 diabetes (Liang et al., 2026). It also participates in the regulation of host lipid metabolism through the synthesis of MCFAs such as caproic acid (Wu et al., 2019; Lee et al., 2020).

In terms of disease association, the abundance of *M. elsdenii* is significantly increased in the intestinal tract of patients with IBD and CRC (Figure 2). A critical unresolved question is the driver/passenger hypothesis: whether elevated *M. elsdenii* abundance directly drives intestinal inflammation and carcinogenesis (driver), or merely proliferates as a secondary consequence of the disordered intestinal microenvironment (passenger). Current evidence supports a context-dependent dual role (Hou et al., 2025; Liang et al., 2026): under homeostatic conditions, it acts as a commensal SCFA producer that sustains epithelial function and metabolic health; when the intestinal barrier is damaged and inflammation persists, it functions as a conditional pathogenic driver. Mechanistically, *M. elsdenii* and its lipopolysaccharide activate dendritic cells (DCs) via the TLR4/NF-κB/IRF4 signaling pathway, induce Th1/Th17 inflammatory responses, and further disrupt intestinal epithelial homeostasis by downregulating tight-junction proteins including Zo-1 and Occludin (Wang et al., 2025). This pathogenic cascade has been validated in AOM/DSS-induced colitis-associated cancer (CAC) mouse models, where *M. elsdenii* colonization directly exacerbates intestinal inflammation and tumorigenesis. In contrast, its abundance is significantly decreased in patients with constipation-predominant irritable bowel syndrome (IBS-C), suggesting its potential involvement in the regulation of intestinal motility (Wang et al., 2025). In addition, abnormal abundance of this bacterium is associated with metabolic and neurological diseases including polycystic ovary syndrome with comorbid depression and cerebral vasculopathy (Liu et al., 2023; Yu et al., 2024). For immunomodulation, this bacterium can induce the maturation of DCs and the secretion of pro-inflammatory cytokines during vaginal dysbiosis, participating in the inflammatory process of bacterial vaginosis (BV) (van Teijlingen et al., 2020). The plasmalogens synthesized by it can enhance the oxidative stress resistance of host cells and indirectly regulate the immune homeostasis of the intestinal mucosa (Zhang et al., 2023).

### 4.3 Functional performance in other mammals

In non-ruminant mammals such as pigs and mice, this bacterium is mainly involved in the regulation of intestinal fermentation and health maintenance, with its effects highly correlated with dietary structure (Figure 2). In piglets, combined use with lactic acid bacteria significantly alleviates weaning stress, reduces intestinal lactate accumulation, increases butyrate production, improves intestinal mucosal morphology, optimizes intestinal microecological structure, and lowers the risk of infection by pathogenic *E. coli* (such as *E. coli* F17) (Yoshida et al., 2009; Sun et al., 2022; Zhang et al., 2023; Yoshikawa et al., 2024). Supplementation with this bacterium also improves protein digestibility, feed conversion ratio, and carcass quality of finishing pigs, and reduces intestinal flatulence (Mølbak et al., 2007; Khorasani et al., 2023). In mouse model studies, this bacterium can correct the intestinal hyperlactatemia induced by fructooligosaccharides (FOS), restore intestinal SCFAs levels, and promote the proliferation of cecal epithelial cells (Hashizume et al., 2003). It also exerts anti-inflammatory effects via the TGF-β signaling pathway and prolongs the lifespan of *Caenorhabditis elegans* (Kwon et al., 2018), indicating its potential probiotic properties. However, in a colitis-associated tumor model, this bacterium significantly exacerbated intestinal inflammation and tumorigenesis (Hou et al., 2025), directly verifying its bidirectional functional potential.

### 4.4 Potential pathogenicity and adverse effects

The symbiotic property of *M. elsdenii* is condition-dependent, and it can exhibit pathogenicity and adverse effects under specific circumstances such as host immunocompromise and intestinal microecological imbalance. This bacterium is a rare opportunistic pathogen: its cell wall lipopolysaccharide (LPS) has low-level endotoxicity, and there have been case reports of it causing human infective endocarditis (Brancaccio and Legendre, 1979; Nagaraja et al., 1979). Recent clinical sequencing and mechanistic studies further confirm that *M. elsdenii* overgrowth is closely associated with refractory intestinal inflammation, precancerous lesion progression, and colitis-associated carcinogenesis in immunocompromised individuals and patients with dysbiotic gut microbiota (Hou et al., 2025; Kubba et al., 2026). During intestinal microecological imbalance, its excessive proliferation can exacerbate the progression of IBD, CRC, metabolic syndrome and other diseases through abnormal accumulation of metabolites and activation of pro-inflammatory pathways (Wu and Park, 2022; Hou et al., 2025). Beyond intestinal diseases, systemic dissemination of *M. elsdenii*-derived outer membrane vesicles (OMVs) may trigger distant inflammatory responses via the gut–lung axis, indicating broader pathogenic risks beyond the gastrointestinal tract (Kubba et al., 2026). In addition, in ruminants, this bacterium may participate in the progression of MFD through the production of specific conjugated linoleic acid (CLA), while its causal relationship and regulatory mechanism have not yet been clarified (Kim et al., 2002; Weimer et al., 2015).

## 5 Industrial application potential

With its high-efficiency carbon source conversion capacity and unique anaerobic metabolic characteristics, *M. elsdenii* can directionally synthesize SCFAs/MCFAs, biohydrogen, and high-value metabolic intermediates, presenting significant industrial development potential in the field of green biomanufacturing. Its ambient-temperature anaerobic fermentation feature can greatly reduce production energy consumption.

In the field of organic acid biosynthesis, this bacterium is a core candidate strain for the green production of SCFAs and MCFAs. The type strain NCIMB 702410 achieves a caproic acid yield of up to 28.42 g/L with a productivity of 0.20 g/(L·h) using sucrose as the carbon source in a two-phase extractive fermentation system, laying a solid foundation for industrial scale-up (Choi et al., 2013). It can adapt to low-cost substrates including Jerusalem artichoke, sweet potato, and agricultural wastes (such as alfalfa juice). The regulation of substrate ratio can increase caproic acid production by 30–50%, with simultaneous co-production of high-value organic acids such as propionate and butyrate (Weimer and Digman, 2013; Wu et al., 2019; Kim et al., 2020), giving it more prominent environmental and cost advantages over traditional chemical synthesis.

In the field of bioenergy, this bacterium realizes efficient hydrogen production via lactate-driven dark fermentation, with a hydrogen yield of 0.95–1.49 mol H_2_ / mol glucose when co-cultured with lactic acid bacteria (Ohnishi et al., 2022). This trait addresses the intractable bottleneck of lactate accumulation-mediated inhibition in biological hydrogen production. It is compatible with raw materials including lignocellulosic biomass and food processing organic wastewater, realizing simultaneous wastewater reclamation and efficient removal of chemical oxygen demand (COD) (Shetty et al., 2013; Caserta et al., 2018; Ohnishi et al., 2022).

In the field of bio-based materials, its encoded propionyl-CoA transferase (Me-PCT) can provide key monomers (such as lactyl-CoA and 3-hydroxybutyrate) for the synthesis of bioplastics including polyhydroxyalkanoates (PHA) (Zhang et al., 2019). 3-hydroxycaproic acid generated via its reverse β-oxidation pathway can be used for the modification of high-performance PHA (Wu et al., 2019; Lee et al., 2020), endowing it with remarkable metabolic engineering potential to meet the development needs of green materials. Optimization of metabolic flux through genome editing can improve the conversion rate of precursor substances, and recombinant *E. coli* strains have been constructed to achieve efficient synthesis of lactyl-CoA at present (Li et al., 2016).

In addition, this bacterium has been commercially applied as a probiotic feed additive for ruminants on a large scale, which can significantly reduce the incidence of ruminal acidosis and improve feed conversion rate (DeClerck et al., 2020; Susanto et al., 2023). For organic wastewater treatment, it can perform fermentative acid production using food processing wastewater (such as lactate-containing wastewater), realizing simultaneous wastewater purification and resource recovery with a COD removal rate of over 60% (Ohnishi et al., 2022). Furthermore, the hydrogenase (MeHydA) derived from this bacterium has modification potential for improved oxygen tolerance, and can be used as a catalytic component for biofuel cells (Caserta et al., 2018).

A critical challenge limiting the industrial translation of *M. elsdenii* as a robust biomanufacturing chassis is the strong cytotoxicity of medium-chain fatty acids (MCFAs, e.g., caproic, caprylic, and valeric acids) during high-titer fermentation. MCFAs damage *M. elsdenii* cells by disrupting cytoplasmic membrane integrity and fluidity, elevating non-specific permeability, inducing intracellular acidification via dissipating transmembrane proton gradients, and inhibiting key enzymes in lactate catabolism and fatty acid synthesis; these effects jointly reduce cell viability, substrate utilization efficiency, and fermentation stability while severely limiting final product titers (Choi et al., 2013; Kim et al., 2020). Based on the core metabolic pathways of *M. elsdenii* outlined in Section 3, targeted engineering strategies can be implemented to enhance MCFA tolerance, including remodeling the reverse β-oxidation pathway to accelerate MCFA synthesis and extracellular efflux, redirecting electron flow by modifying the EtfAB-mediated electron-bifurcation pathway to sustain intracellular redox homeostasis (Nguyen et al., 2026), strengthening the lactate-acrylate metabolic shunt to alleviate MCFA overproduction, engineering membrane stability and MCFA-specific efflux pumps to mitigate membrane damage, and heterologously expressing stress resistance modules to counteract protein denaturation and reactive oxygen species accumulation. These pathway-guided engineering strategies are essential for upgrading *M. elsdenii* from a laboratory-scale strain to a commercially viable industrial biomanufacturing chassis by resolving the core bottleneck of MCFA toxicity in industrial scale-up fermentation.

## 6 Potential risks and controversies

**Figure 3:**
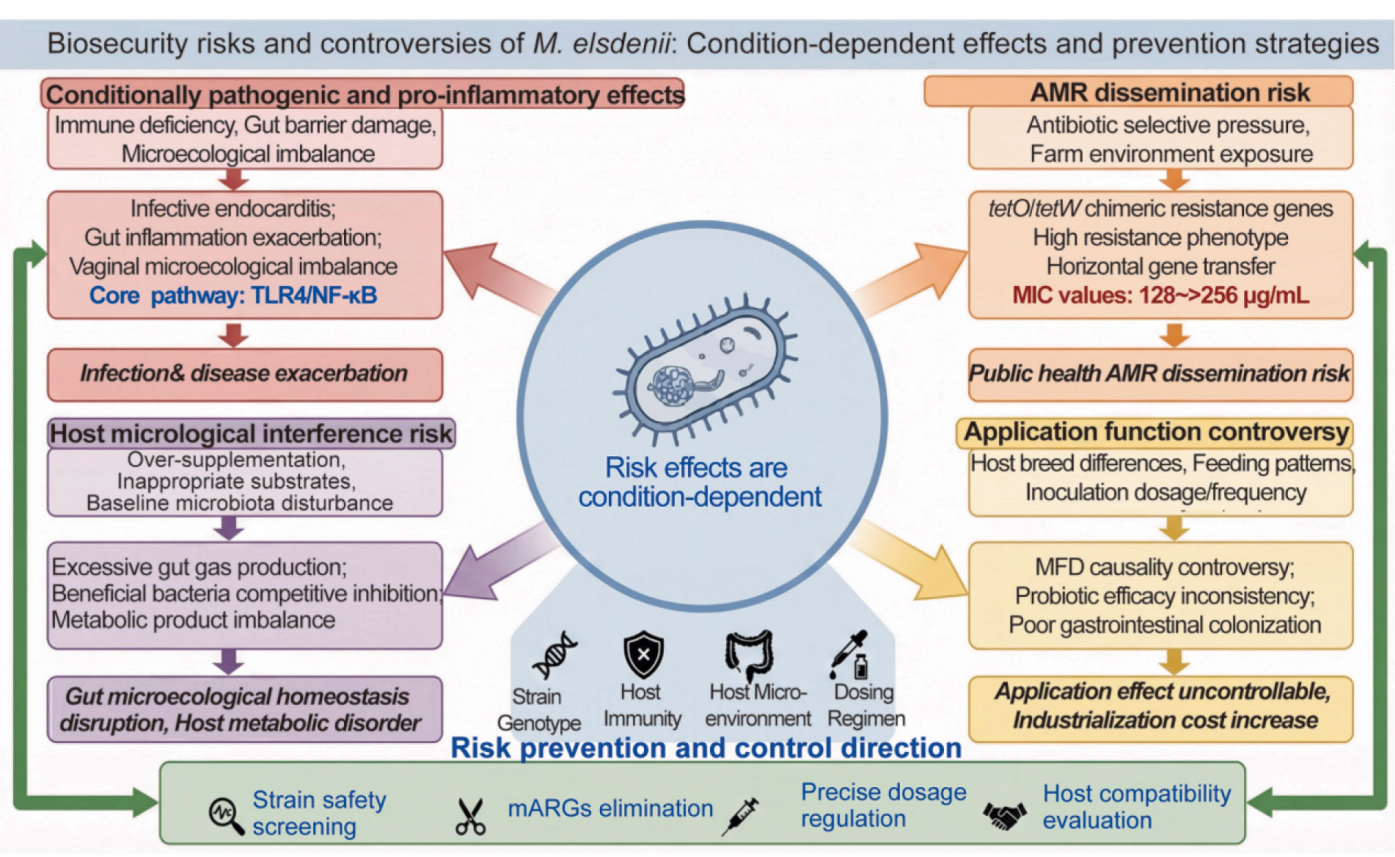
Biosecurity risks and mitigation of *M. elsdenii*: Condition-dependent effects. This figure illustrates the four core biosafety risks of *M. elsdenii* and their regulatory factors, as well as the specific physiological contexts that drive risk exacerbation: (1) Conditional pathogenicity is triggered by host immunocompromise, impaired intestinal barrier integrity, and sustained gut microecological dysbiosis, which enable *M. elsdenii* to act as a conditional driver of intestinal inflammation, colitis-associated carcinogenesis, vaginal dysbiosis, and rare systemic infections such as infective endocarditis (Brancaccio and Legendre, 1979; van Teijlingen et al., 2020; Hou et al., 2025); (2) AMR dissemination is driven by long-term antibiotic selective pressure in livestock farming, during which *tet(O)/tet(W)* chimeric resistance genes are horizontally transferred among gut commensals via mobile genetic elements, posing a public health hazard (Stanton and Humphrey, 2003; Stanton et al., 2004); (3) Application functional controversies include strain-specific probiotic efficacy variation and debated contributions to ruminant milk fat depression (Weimer et al., 2015; Cabral and Weimer, 2024); (4) Host microecological disturbance arises from high-dose excessive supplementation, unmatched carbon substrates, and unstable gastrointestinal colonization, causing gas accumulation, beneficial microbiota inhibition, and metabolic imbalance (Rose et al., 2021; Mutuyemungu et al., 2024). Effective risk mitigation relies on stringent safety screening of candidate strains, host-adapted application evaluation, precise dose control, and targeted removal of ARGs, and avoidance of overproliferation in immunodeficient or inflammatory host backgrounds (Huertas-Díaz et al., 2025; Strachan et al., 2025).

*M. elsdenii* exhibits a distinct dual nature in terms of application potential and biological safety, with its potential risks mainly concentrated in four dimensions: opportunistic pathogenicity, AMR dissemination, functional controversies, and host microecological disturbance (Figure 3). The core of relevant controversies stems from the genotypic specificity of strains, differences in host microenvironments, and the rationality of application scenarios.

This bacterium is a typical opportunistic pathogen. In rare cases, it can cause human infective endocarditis, and its cell wall LPS has low-level endotoxicity (Brancaccio and Legendre, 1979; Nagaraja et al., 1979). Its abundance is abnormally elevated in the intestinal tract of patients with IBD and CRC, where it can activate Th1/Th17 pro-inflammatory responses via the TLR4/NF-κB signaling pathway and disrupt intestinal epithelial homeostasis (Hou et al., 2025; Wang et al., 2025). It can also exacerbate the local inflammatory process of BV (van Teijlingen et al., 2020).

AMR dissemination is its core public health hazard. Swine-derived strains generally carry *tetO*, *tetW* and their chimeric tetracycline resistance genes, with a MIC ranging from 128 to >256 μg/mL, and 7 resistance genotypes have been identified to date. As an intestinal symbiont, its ARGs can spread between hosts and among intestinal flora via horizontal gene transfer, aggravating the risk of AMR dissemination (Stanton and Humphrey, 2003; Stanton et al., 2004, 2011; Piknová et al., 2006).

In terms of application, there are still significant controversies regarding the effect of this bacterium on milk fat metabolism in dairy cows and the host specificity of its probiotic efficacy. In addition, it is difficult for the strain to colonize the gastrointestinal tract for a long time after a single inoculation, requiring repeated supplementation (Weimer et al., 2015; Lopes et al., 2021; Cabral and Weimer, 2024; Francis et al., 2024). Inappropriate supplementation may also induce host intestinal microecological disorders, including intestinal flatulence caused by excessive gas production (Rose et al., 2021; Mutuyemungu et al., 2024), competitive inhibition of beneficial bacteria (such as inhibited growth of *Bifidobacterium* and *Enterococcus faecalis*) (Hashizume et al., 2003; Yoshida et al., 2009), and lipid metabolism disturbance triggered by metabolite imbalance (e.g., excessive production of MCFAs such as valeric acid and caproic acid) (Wu and Park, 2022; Huertas-Díaz et al., 2025).

## 7 Research gaps and future directions

Despite the promising potential of *M. elsdenii* as a gastrointestinal symbiont and industrial chassis, critical research gaps remain that restrict its fundamental mechanistic understanding and practical translation.

First, a mature native genetic manipulation system is entirely lacking, with no validated transformation protocols, adapted CRISPR–Cas editing tools, or characterized regulatory element libraries available, severely limiting targeted metabolic engineering. Second, the context-dependent dual role (commensal vs. pathogen) and driver/passenger hypothesis in intestinal diseases remain unresolved, as clinical and causal evidence distinguishing direct carcinogenic effects from secondary overgrowth is insufficient. Third, MCFA cytotoxicity and low product tolerance represent major bottlenecks for industrial scale-up, and rational engineering strategies tailored to its core metabolic pathways remain underexplored. Fourth, biosafety risks—including conditional pathogenicity, horizontal transfer of antibiotic resistance genes, and gut microecological disturbance—lack systematic assessment standards and risk-control solutions. Fifth, interspecies cross-feeding, host-specific adaptation, and distal physiological effects (e.g., gut–lung axis) mediated by OMVs remain largely uncharacterized.

Future research should focus on five core directions: developing native genetic tools and high-efficiency genome-editing systems; validating the driver/passenger role and molecular mechanisms in intestinal inflammation and tumorigenesis; engineering MCFA tolerance and optimizing scaled fermentation; establishing biosafety evaluation and risk-mitigation frameworks; and dissecting host–microbe and microbe–microbe interactions across host species and mucosal niches. These advances will unlock the full potential of *M. elsdenii* for clinical, agricultural, and industrial applications.
